# Associations of cardiovascular risk factors with handgrip strength and gait speed among older males and females: A systematic review protocol

**DOI:** 10.1371/journal.pone.0344309

**Published:** 2026-03-13

**Authors:** Allen Suzane de França, Amanda Silva Saldanha, Alex Moreira da Silva, Catherine M Pirkle, Ana Tereza do Nascimento Sales Figueiredo Fernandes, Saionara Maria Aires da Câmara

**Affiliations:** 1 Postgraduate Program in Physiotherapy, Federal University of Rio Grande do Norte, Natal, Rio Grande do Norte, Brazil; 2 Department of Physiotherapy, Federal Federal University of Rio Grande do Norte, Natal, Rio Grande do Norte, Brazil; 3 Office of Public Health Studies, University of Hawaiʻi at Mānoa, Honolulu, Hawaii, United States of America; 4 Department of Physiotherapy, State University of Paraíba, Campina Grande, Paraíba, Brazil; Japanese Red Cross Medical Center, JAPAN

## Abstract

**Introduction:**

Cardiovascular risk factors (CRFs), such as hypertension, diabetes, dyslipidemia, and smoking are associated with several adverse health outcomes among older people, such as poor physical function. Handgrip strength and gait speed are key measures of physical function that indicate general health status among older people. Nevertheless, it is unclear which factors have the greatest impact on these measures in older adults. In addition, gender differences in cardiovascular disease risk are observed, with older women with a history of diabetes, obesity, and hypertension being more prone to cardiovascular events than older men. This may be related to different patterns of cardiovascular aging, which may impact the physical function of older men and women differently over time. This article aims to describe the protocol for a systematic review to identify which CRFs are associated with measures of physical function (handgrip strength and gait speed) and whether this association varies by sex.

**Methods and analysis:**

A systematic search will be conducted in the PubMed, LILACS (Latin American and Caribbean Health Sciences Literature), Cochrane Library, Web of Science and Embase databases. The sorting and tracking of articles will be performed with the help of the Rayyan platform. Following the recommendations of the Preferred Reporting Items for Systematic Review and Meta-Analysis Protocols (PRISMA-P), the main reviewer will conduct the initial search and exclusion of duplicates, and two researchers will extract data from the selected articles independently and blindly, using an instrument built specifically for this research. Discrepancies will be solved by a third reviewer. Cross-sectional and/or longitudinal studies, involving community-dwelling older individuals and that have assessed the association between CRFs and measures of physical function (handgrip strength and/or gait speed) will be included. The methodological quality of the included studies will be assessed using the Quality Assessment Tool for Observational Cohort and Cross-Sectional Studies.

**PROSPERO registration number:**

CRD420251167299.

## Introduction

Population aging is a global phenomenon marked by the increased prevalence of chronic non-communicable diseases, representing the greatest burden on global health [1]. Some of these chronic conditions, such as diabetes and hypertension, increase the risk of serious cardiovascular events, such as acute myocardial infarction and stroke, which remain to be the main cause of death in the world [2,3]. In addition to being responsible for high costs to public health systems, they contribute significantly to an increase in years lived with disability [3].

It has been suggested that some CRFs such as hypertension, hypercholesterolemia and diabetes mellitus tend to accumulate in older adults, which can negatively affect physical function over time [4,5]. Previous studies demonstrated a strong association between diabetes and functional limitation and disability in older adults [6]. In addition, hypertensive individuals have a greater risk of experiencing a decline in physical function [7].

The objective tests of physical function, such as gait speed or hand grip strength test, are important predictors of poor health outcomes, such as disability and premature mortality [8,9]. They also play key roles in diagnosing and monitoring important age-related conditions such as sarcopenia and frailty [10,11]. Maintaining physical function is crucial for a better quality of life and independence for the older people, in addition to being considered an important protective factor for adverse health events such as falls, hospitalizations and death [12–15].

Although there is evidence of an association between some CRFs and physical function [16–18], it is not clear which factors more strongly impact measures such as handgrip strength and gait speed among older people. Understanding these associations could help to clarify etiology pathways of functional decline, identify those at higher risk of disability, and support preventive health strategies. Furthermore, it has been demonstrated that there are sex differences in the relative risks of traditional CRFs [19]. Indeed, the presence of diabetes, obesity and hypertension in women, particularly those over 60 years of age, represents a higher risk for developing cardiovascular diseases (CVD) compared to men with the same risk factors [20–23]. Women with diabetes have a higher incidence of CVD-related mortality, myocardial infarction, heart failure and stroke than men with diabetes [24]. It has also been reported that women and men present distinct patterns of cardiovascular aging [25]. Thus, it is possible that CRFs impact physical function of older men and women differently over time.

The objective of this paper is to describe a protocol for a systematic review that aims to identify which CRFs are associated with physical function (handgrip strength and gait speed) among older people and to verify if the associations vary according to sex.

For this review, two research questions will be asked: “Which CRFs are associated with handgrip strength and gait speed in community-dwelling older adults?” and “Are there sex differences in the association between cardiovascular risk factors and these measures of physical function?”.

## Methods and analysis

### Registry

This systematic review protocol follows the Preferred Reporting Items for Systematic Review and Meta-Analysis Protocols (PRISMA-P) guide [26] (S1 Checklist). It was registered in the International Prospective Register of Systematic Reviews (PROSPERO) with registration number CRD420251167299 in October 2025.

### Eligibility criteria

**Types of study.** Papers published in peer-reviewed journals, which have an observational design, whether cross-sectional or longitudinal, and that aimed to investigate the association between CRFs and hand grip strength and/or gait speed, in community-dwelling older adults will be included. There will be no limitations regarding language, country or year of publication. If any of the selected papers are not in Portuguese, English, French or Spanish, they will be sent to a translation service.

**Types of participants.** The review will examine studies reporting on community-dwelling older males and/or females (60 years or older for studies conducted in low- or middle-income countries and 65 year or older for those conducted in high-income countries). According to The World Bank, low- and middle-income countries are defined as those with a Gross National Income (GNI) per capita of $1,145 or less in 2023, and $1,146 and $4,515, respectively, for the current 2025 fiscal year. While high income countries are those with more than a GNI per capita of $14,005 [27]. Studies that only include institutionalized older people will be excluded. Institutionalized older people are more likely to have serious adverse health conditions that can confound the associations between CRFs and physical function. Studies involving exclusively older people with specific health conditions, such as cancer, hip fracture, osteoarthritis, will not be included because they may influence the associations between CRFs and physical function.

**Exposure.** The CRFs considered in the calculation of the Framingham risk score will be considered as exposure, which includes age, total cholesterol, HDL-cholesterol levels, systolic blood pressure, smoking and diabetes status [28]. Both single and combined CRFs that have been identified objectively (such as through blood exams and physical assessments) or through self-report (use of medications or previous diagnosis by a health professional) will be analyzed. Quantitative information will also be collected when available, such as the exact number of cigarettes smoked per day, blood pressure millimetres of mercury (mmHg), and specific concentrations of biochemical markers.

**Primary outcomes.** The outcome variable of this study will be measures of physical function (handgrip strength and/or gait speed).

### Search

**Search strategy.** The search for articles will be carried out in the PubMed, Web of Science, LILACS (Latin American and Caribbean Health Sciences Literature), Cochrane library and Embase databases. Additionally, a manual search will be performed using the bibliographic references of the included articles. The search strategy considers the Medical Subject Headings (MeSH terms) and keywords related to the topic. The descriptor related to CRFs and similar terms was combined with the descriptors of physical function, older people, and community dwelling population. Initially, a search strategy was developed in PubMed using MeSH terms, and then terms related to the research question were added. Adaptations were made for the search in other databases (S1 Appendix).

**Selection of relevant studies.** The main reviewer (ASdF) will carry out the first search in the selected databases applying the eligibility criteria and the selected articles will be inserted into the Rayyan platform (https://www.rayyan.ai/). After removing duplicates, two independent researchers (ASS and AMdS) will select the studies independently of each other by reading the titles and abstracts. Then, they will read the remaining articles in full and will check if they meet the eligibility criteria. If there are discrepancies between the selected articles, a separate researcher will resolve them (ASdF).

### Data extraction

The main researcher (ASdF) will be responsible for extracting data from the selected articles using a tool specifically designed for this research. A second researcher (ASS) will independently and blindly review a random set of 10% of the articles and the inter-rater reliability (IRR) will be assessed. If the IRR is high, the main researcher will complete the data extraction alone. Otherwise, the two investigators will jointly review the data extraction procedures and expectations and then attempt a random selection of 10% again.

The following data will be collected: general study information (main author, title; main goal; year, country, journal of publication; original language), information regarding the methods (sample size; average age and age range of the study sample; number and proportion of men and women; study design; cardiovascular risk variables assessed; instruments used to assess physical function; covariates considered, with special attention to respiratory comorbidities and lifestyle factors), results (relationships found between CRFs and measures of physical function, including measures of association and that statistical significance of the results, and sex differences if assessed). Additionally, information regarding female-specific cardiovascular risk factors will be extracted whenever available. These include menopausal status (age at onset and type of menopause), history of pregnancy-related complications (such as gestational diabetes and pre-eclampsia), and presence of ovarian disorders (e.g., polycystic ovary syndrome).

### Methodological quality assessment

The Quality Assessment Tool for Observational Cohort and Cross-Sectional Studies will be applied by the same reviewers as in the previous phase to evaluate methodological quality of the selected studies. A third researcher (ASS) will resolve the discrepancies if any arise.

### Data synthesis

A flowchart adapted from PRISMA 2020 [29] will be created to demonstrate the study selection process, from identification to final inclusion of articles.

The extracted data will be presented in tables and will be grouped according to the type of CRFs. Tables will be constructed with the variables associated with each of the physical function tests, handgrip strength and gait speed, separately. To compare different evaluation items, gait speed and handgrip strength results will be converted to a common metric (meters per second, m/s, and kilogram-force, kgf) whenever possible. Tables with the association data separated by sex will also be presented. If it is possible to perform a meta-analysis, the RevMan 5 program will be used [30]. For handgrip strength, where different dynamometers may be used, we will employ the Standardized Mean Difference (SMD) in the meta-analysis to allow for cross-study comparison. This approach will also be applied to gait speed to account for variations in protocols, such as different walking distances or timing methods. Heterogeneity will be assessed using the I^2^ statistic. Given the anticipated diversity in study populations and settings, a random-effects model will be utilized to provide a more conservative and generalizable estimate. To further investigate sources of heterogeneity, subgroup analyses will be conducted based on: [1] Country income level (High vs. Low/Middle income); [2] Sex (Male vs. Female); and [3] Assessment method (Objective vs. Self-reported CRFs). To explore the influence of biological sex more deeply, a sensitivity analysis will be conducted within the female subgroup. We will compare results from studies that account for female-specific risk factors (such as menopausal status or history of pre-eclampsia/gestational diabetes) against those that only report traditional CRFs. This will allow us to assess whether these reproductive and hormonal factors significantly alter the association between cardiovascular risk and physical function (handgrip strength and gait speed). A 95% confidence interval and p-values will be considered in these analyses.

### Study timeline

To complete all stages of the systematic review, including the selection of relevant studies, data extraction, quality assessment, and data synthesis, we anticipate a timeframe of 8–10 months.

### Patient and public involvement

There was no participation of patients and/or the public at any stage of this protocol.

### Ethics and disclosure

This systematic review does not require ethical approval. The review results will be shared with the scientific community in peer-reviewed journals, relevant academic events, and at meetings with geriatric and cardiovascular health professionals.

## Discussion

This systematic review will enable a comprehensive and accurate analysis of the available evidence on the cardiovascular risk factors that impact the physical function of older adults, particularly those that affect men and women differently. By gathering and critically evaluating the data, this study aims to identify which specific CRFs, and possibly combinations thereof, that are most strongly associated with measures of physical function.

Given the multifactorial nature of cardiovascular and metabolic diseases, their impact cannot be fully understood when factors are analyzed in isolation. Furthermore, although composite risk scores are widely used to predict cardiovascular events, they may obscure the individual contributions of each component when the outcome of interest is musculoskeletal impairment rather than cardiovascular disease itself. Recognizing the cumulative and individual impact of these factors will provide robust evidence for the early identification of vulnerable individuals, potentially guiding targeted rehabilitation and prevention strategies.

An important aspect of this research is the analysis of gender heterogeneity in the manifestation and impact of these risk factors on physical function. It is hypothesized that biological differences in the aging process between men and women, notably the more pronounced cardiovascular and endocrine changes in aging women, may result in unequal physical function outcomes, with worse results for older women.

By consolidating knowledge about these associations and the differences found according to gender, the findings of this review are expected to inform clinical decisions and public health. The expected results may contribute to the development of health policies and interventions such as improving risk screening tools, which will consequently contribute to improving the quality of life and mitigating functional decline in older adults.

### Strengths and limitations

This systematic review will gather relevant information on the association between cardiovascular risk factors and measures of physical function in older adults, and may also identify differences according to gender.

Considering gender differences in the analyses and knowing that there are female-specific cardiovascular risk factors, such as menopause, this data may not be available in the articles to be included in this systematic review, or may have been assessed inconsistently in observational studies, which may limit the understanding of cardiovascular risk factors that may impact the physical function of older men and women specifically.

Additionally, heterogeneity of instruments for assessing physical function, of criteria for identifying cardiovascular risk factors, and of confounding variables can limit quantitative analyses.

## Supporting information

S1 Checklist PRISMA-P Checklist. (DOCX)

S1 Appendix Search strategy for according to databases. (DOCX)
