# Supplementary material for: Associations of cardiovascular risk factors with handgrip strength and gait speed among older males and females: A systematic review protocol
Source: PLoS One. 2026 Mar 13;21(3):e0344309. doi: 10.1371/journal.pone.0344309 (PMC12987416; doi:10.1371/journal.pone.0344309)
Supplement: S1 Appendix — (DOCX) [file pone.0344309.s002.docx]

**S1 Appendix**

| **Database** | **Search Terms** |
| --- | --- |
| PubMed/MEDLINE  http://www.ncbi.nlm.nih.gov/pubmed/ | ("Cardiovascular Diseases/epidemiology"[MeSH Terms] OR "cardiovascular risk factors" OR "cardiovascular risk burden") AND ("Aged"[MeSH Terms] OR "older adult*" OR "elderly people" OR "older people") AND ("handgrip strength" OR "gait speed" OR "walking speed" OR "daily life activities" OR "Activities of Daily Living" OR "disability" OR "functional capacity" OR "Mobility Limitation") |
| Web of Science by CAPES  https://www-webofscience-com.ez18.periodicos.capes.gov.br/wos/woscc/basic-search | ALL=("cardiovascular risk factors" OR "cardiovascular risk burden") AND ALL=("older adults" OR "elderly" OR "aged" OR "older people") AND ALL=("handgrip strength" OR "gait speed" OR "walking speed" OR "activities of daily living" OR "daily life activities" OR "disability" OR "functional capacity" OR "Mobility Limitation") |
| LILACS by BVS  https://bvsalud.org/ | ((cardiovascular risk factors) OR (cardiovascular risk burden) ) AND ((aged) OR (older adult*) OR (elderly people) OR (older people)) AND ((grip strength) OR (handgrip strength) OR (gait speed) OR (walking speed) OR (daily life activities) OR (activities of daily living) OR (disability) OR (functional capacity) OR (mobility limitation)) AND db:("LILACS") AND instance:"lilacsplus" |
| COCHRANE  https://www.cochranelibrary.com | ((Cardiovascular Diseases/epidemiology OR cardiovascular risk factors OR cardiovascular risk burden) AND (Aged OR older adult OR elderly people OR older people) AND (handgrip strength OR gait speed OR walking speed OR daily life activities OR Activities of Daily Living OR disability OR functional capacity OR Mobility Limitation)) in Title Abstract Keyword |
| EMBASE by CAPES  https://www-embase-com.ez18.periodicos.capes.gov.br/search/quick?phase=continueToApp | ('cardiovascular risk factor'/exp OR 'cardiovascular risk factors' OR 'cardiovascular risk burden') AND ('aged'/exp OR 'aged' OR 'older adult*' OR 'elderly' OR 'older people'/exp OR 'older people') AND ('grip strength'/exp OR 'handgrip strength' OR 'walking speed'/exp OR 'gait speed' OR 'daily life activity'/exp OR 'activities of daily living' OR 'disability'/exp OR 'functional status'/exp OR 'functional capacity' OR 'walking difficulty'/exp OR 'mobility limitation') AND [embase]/lim |
